# RF Path and Absorption Loss Estimation for Underwater Wireless Sensor Networks in Different Water Environments

**DOI:** 10.3390/s16060890

**Published:** 2016-06-16

**Authors:** Umair Mujtaba Qureshi, Faisal Karim Shaikh, Zuneera Aziz, Syed M. Zafi S. Shah, Adil A. Sheikh, Emad Felemban, Saad Bin Qaisar

**Affiliations:** 1 Department of Computer Science, City University of Hong Kong, Kowloon, 852, Hong Kong, China; zaziz3-c@my.cityu.edu.hk; 2 Department of Telecommunication, Mehran University of Engineering and Technology, Jamshoro 76062, Pakistan; faisal.shaikh@faculty.muet.edu.pk (F.K.S.); SyedMZ@cs.cardiff.ac.uk (S.M.Z.S.S.); 3 School of Computer Science & Informatics, Cardiff University, Cardiff CF10 3XQ, UK; 4 Science and Technology Unit, Umm Al Qura University, Makkah 24382, Saudi Arabia; aasheikh@uqu.edu.sa; 5 Department of Computer Engineering, Umm Al Qura University, Makkah 24382, Saudi Arabia; eafelemban@uqu.edu.sa; 6 CoNNekT Lab, National University of Sciences and Technology, Islamabad 44000, Pakistan; saad.qaisar@seecs.edu.pk

**Keywords:** UWSNs, sensors, underwater, water conductivity, water permeability, water permittivity, freshwater, seawater

## Abstract

Underwater Wireless Sensor Network (UWSN) communication at high frequencies is extremely challenging. The intricacies presented by the underwater environment are far more compared to the terrestrial environment. The prime reason for such intricacies are the physical characteristics of the underwater environment that have a big impact on electromagnetic (EM) signals. Acoustics signals are by far the most preferred choice for underwater wireless communication. Because high frequency signals have the luxury of large bandwidth (BW) at shorter distances, high frequency EM signals cannot penetrate and propagate deep in underwater environments. The EM properties of water tend to resist their propagation and cause severe attenuation. Accordingly, there are two questions that need to be addressed for underwater environment, first what happens when high frequency EM signals operating at 2.4 GHz are used for communication, and second which factors affect the most to high frequency EM signals. To answer these questions, we present real-time experiments conducted at 2.4 GHz in terrestrial and underwater (fresh water) environments. The obtained results helped in studying the physical characteristics (*i.e.*, EM properties, propagation and absorption loss) of underwater environments. It is observed that high frequency EM signals can propagate in fresh water at a shallow depth only and can be considered for a specific class of applications such as water sports. Furthermore, path loss, velocity of propagation, absorption loss and the rate of signal loss in different underwater environments are also calculated and presented in order to understand why EM signals cannot propagate in sea water and oceanic water environments. An optimal solk6ution for underwater communication in terms of coverage distance, bandwidth and nature of communication is presented, along with possible underwater applications of UWSNs at 2.4 GHz.

## 1. Introduction

Underwater Wireless Sensor Networks (UWSNs) are comprised of a sensor or a group of sensors that are deployed inside water to sense and explore the deep underwater environments [1]. These sensors communicate wirelessly, with each other and with the central base station where information of different sensors is collected for underwater applications [2]. There are numerous UWSN applications [3] such as underwater monitoring applications (e.g., water quality monitoring, habitat monitoring and deep oceanic exploration), underwater disaster recovery applications (e.g., natural disasters and man-made disasters), underwater military applications (e.g., mines, submarine and surveillance applications), assisted navigation and underwater sports applications. Despite of many UWSN applications, the harsh environment of the underwater environment makes it difficult to design a robust and a reliable network. This is because the underwater environment acts as a channel, and wireless communication through underwater channels has always been extremely challenging. The intricacies presented by the underwater channel are far greater than the problems experienced in terrestrial environment. The prime reason of such intricacies are physical characteristics of underwater channels that tends to allow only restricted frequency bands [4,5], such as acoustic, electromagnetic (EM) and optical bands. To design an efficient and robust UWSN, the characteristics of underwater channels are of prime importance. The study of underwater physical characteristics (e.g., electro-magnetic properties, propagation and absorption loss) will help to highlight the possible effects of on the operating frequencies and design an adaptable UWSN [4]. Therefore, it is important to discuss the underwater channel characteristics in detail and to know the possible effects of channel characteristics on different operating high-frequency bands.

There are different kinds of communication systems for underwater environments. Underwater Acoustic Communication Systems (UW-ACSs) utilize low electro-mechanical frequencies (few Hz) such as acoustics for information transmission from one point to another [6]. Since the frequencies are low (large wave lengths), they are suitable for long distance communication such as ocean depth monitoring and exploration [2,7]. Acoustic waves get severely attenuated by ambient noise and high water turbidity. Generally, the propagation speed of UW-ACS is approximately 1500 m/s, which results in high propagation delays and Bit Error Rate (BER) [8]. Therefore, for real-time applications, UW-ACSs are not considered suitable. Unmanned underwater vehicles (AUVs), buoys and underwater nodes communicate each other using acoustic signals. On the other hand, Underwater Optical Communication Systems (UW-OCSs) use optical frequencies for information transmission [9]. These are typically very high frequencies of the order of THz. UW-OCSs can be used for shallow water applications specifically at less distances (few meters) as light is attenuated highly at greater distances inside water [9]. Optical laser lights are not preferred for long distance applications because UW-OCS requires accurate Line of Sight (LOS), which is extremely difficult to establish and maintain [6]. AUVs and surface stations may communicate over optical links. Underwater Radio Wave Communication Systems (UW-RCSs) operate on radio waves in which the frequencies are generally of the order from kHz to GHz [2]. As compared to UW-ACSs, the UW-RCSs have the luxury of high bandwidth, high data rates and low propagation delays [10]. Generally, radio wave communication is considered for over the water communication between surface stations and buoys, and on shore infra-structure and anchored underwater nodes may communicate over radio links. Furthermore, UW-RCSs can be used for short distance real-time underwater applications and mostly used in shallow water applications [3] as the utilized radio waves do not penetrate deep in water [2]. Due to conductive nature of the water, radio frequencies get severely attenuated [7]. However, unlike other UW communication systems, radio based UW-RCSs are less affected from ambient noise, water turbidity and temperature [11].

**Figure 1:**
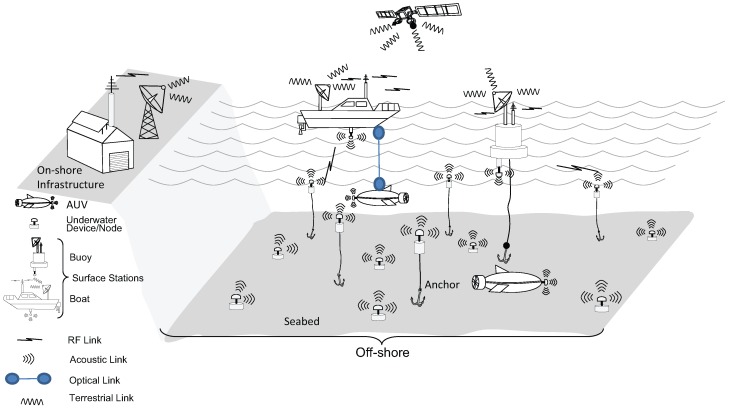
Generic underwater sensor network architecture [3].

Table 1 and Figure 1 summarize the three types of underwater communication systems. These systems are differentiated on the basis of the carrier frequency, bandwidth, transmission distance and nature of communication in underwater. Keeping in view the existing technologies and applications, a trade-off exists between the transmission distance, bandwidth and the nature of communication, and, accordingly, an optimal carrier may be chosen [2].

The rest of the paper is organized as follows. Section 2 provides details of the underwater channel characteristics. Section 3 focuses on possible effects over propagation of high EM signals. Section 4 presents the experiments to calculate path loss in a terrestrial and underwater environment. Furthermore, the section presents a detailed analysis over the path loss profile in the two environments and highlights the key factors. Section 5 concludes the paper.

## 2. Related Work

Generally, UW-WCSs use acoustic signals, which are low frequencies in comparison to the other frequencies used for UW communication. There are many papers discussing the propagation scenarios for acoustic waves in underwater communication and applications [1,2,4,5]. Thus, the challenge is to work on high frequency bands (in the range of GHz) for underwater communication and applications, which is an open area for the researchers. There are many authors who have researched about the medium characteristics and also illustrated the comparative study over the various frequencies used for underwater wireless communication such as acoustic, radio and optical signals in UW environments.

In [6], the authors have analyzed path loss by reflection and refraction of the acoustic signals in underwater environment. In [4], the authors have worked on estimating the effects of depth of water and effects of temperature over acoustic frequencies. The authors in [7] presented a detailed description relating various parameters of high frequency waves (2.4 GHz) such as propagation velocity, total path loss, wavelength and frequency with different values of distance and conductivity of the water medium for UWSNs. They focused on using high frequency EM wave propagation and characterizing the channel with magnetic permeability, di-electric permeability and permittivity, electric conductivity and volume charge density of the water medium.

In [10], the authors performed simulations with 3 kHz for underwater communication and achieved distances of around 40 m between the communicating nodes. In [12], the authors also tested multiple frequencies and estimated maximum distances. At 100, 10 and 1 kHz, the maximum distances traveled were 6, 16 and 22 m, respectively.

In [13], the authors have developed an initial design of Field-Programmable Gate Array (FPGA) based underwater high frequency (ranging from 100 kHz to 1 MHz) acoustic modem for short distance communication. Utilizing the greater bandwidth of this frequency, the authors have been able to achieve substantially better results for shorter distances in terms of symbols per signal. The modem employed Binary Phase-Shift Keying (BPSK) modulation, with a transmission rate of 1 kbps of data rate with a duration of around 1 ms and a wait time of 20 ms. They stated that it is an appropriate interval to avoid the effects of reflections under water.

In [14], the authors discouraged the use of RF band for UW communication and have used the Very Low Frequency (VLF) band of the electromagnetic spectrum as it does not suffer more attenuation as compared to other higher frequencies. The authors stated that the high attenuation is due to the conductive water medium. In addition, they reported 2.4 GHz frequency band as an invalid band for UW communications. Though high frequency EM signals do offer higher throughput as compared to acoustic signals, but the order of attenuation is very severe in UW communication systems.

Much less research has been done on estimating underwater transmission loss or path loss at 2.4 GHz [15]. Therefore, this paper focuses on estimating path loss for 2.4 GHz frequency band for UW-WCSs. We further extend our work to different UW environments, absorption loss estimation and possible effects of water conductivity over 2.4 GHz bands. By transmitting at 2.4 GHz in UW environments, this paper justifies the use of 2.4 GHz bands in certain scenarios rather than proclaiming it to be a totally invalid band for UWSNs like in [14] and also proposes underwater applications using 2.4 GHz.

## 3. Underwater Channel Characterization

In order to design and deploy any UWSN applications, we need to decide the carrier frequency and, more importantly, the factors that influence the carrier frequency. To illustrate the factors, the 2.4 GHz ISM license free frequency band is considered for underwater wireless communication. There are a number of factors that influence the EM wave propagation underwater. These factors are medium and frequency specific. As these factors are analyzed, a relationship between the UW wireless channel and free space practical models can be built. This section demonstrates channel characteristics and explains the influence over the carrier frequency in the UW medium.

### 3.1. Path Loss

Path loss is a major channel characteristic for any medium that is considered for designing an efficient and robust UW system. Whenever an EM wave propagates in any channel (UW or free space), it degrades with an increase in the distance. The amount of degradation over a particular distance from the transmitter to the receiver is indicated from a term called Path Loss Attenuation or Path Loss. The factors that cause path loss are channel and frequency specific. In free space, the factors that cause path loss are diffraction, reflection, free-space loss or coupling losses, and terrain contours from different environments (urban or rural) [16]. It also gets affected by vegetation and foliage, propagation medium (dry or moist air), the distance between the transmitter and the receiver, and the height and location of antennas [16]. For underwater medium, the factors causing path loss for EM waves are the EM properties of water, multipaths, absorption losses, *etc.* These factors act differently over different frequency bands in a UW channel. In both scenarios, the relationship between signal power and distance is shown in Equation (1) [16].

(1) $$P_{r}\propto d^{-n}$$

where $P_{r}$ is a received signal power, *d* is the distance, and *n* is the path loss exponent. The relation states the simultaneous decrease in signal power as it propagates with respect to distance.

The factor that characterizes path loss in different environments is called Path Loss Exponent. The path loss exponent characterizes the loss of signal strength when it propagates in different environments [16].

Table 2 shows the values of the path loss exponent for different environments. A higher value of *n* characterizes lossy environments [16]. In some environments, such as buildings, stadiums and other indoor environments, the value of *n* ranges between 4 and 6 [16]. On the other hand, a tunnel may act as a waveguide, resulting in a path loss exponent less than 2. For a UW environment, which is a harsh environment, the path loss exponent ranges between 2 and 4 [17]. Depending on the type of environment for communication; various models have been designed to calculate the path loss. In our case, we use the Log Distance Model [16] to estimate path loss by calculating Received Signal Strength (RSS) in Equation (2) at different distances in UW environments.

(2) RSS=10×n×log(d)+C

where RSS is the loss in dBs, *n* is the path loss exponent; *d* is the separation distance between transmitter and receiver usually in meters, and *C* indicates the system losses.

In order to calculate the path loss exponent in any environment, the strength of the signal needs to be estimated at a particular power from different discrete distance points. This will give an overall approximation of how signal strength decreases with increasing distance in that particular environment. Since power of signal decreases with increasing distance, the factor *n* will be a linear regression line [18]. As in Equation (3),

(3) y=m×d+C

where *y* is a straight line, *m* is the slope of the line, *d* is the distance along which the values are calculated, and *c* is constant. Using Equation (2) and Equation (3), we can extract the path loss exponent as n=m/10. This relation will be used to estimate the path loss exponent of both of the environments, *i.e.*, UW and terrestrial indoor environments.

### 3.2. Electro-Magnetic Properties of Water

The high frequency radio band facilitates the network with large bandwidth for high data rate applications. However, the disadvantage is that EM waves attenuate severely underwater and thus are not able to travel long distances. In order to realize the possible effects on EM waves that propagate wirelessly underwater, the following EM properties of water are to be considered [11,18].

#### 3.2.1. Magnetic Permeability of the Medium

The magnetic permeability is the ability of a medium to store magnetic fields [11]. As water and air, both are non-magnetic in nature, and their relative permeability is same [11,19]. Thus, permeability of water has no effect over the EM propagation.

#### 3.2.2. Dielectric Permittivity of the Medium

The relative permittivity (*μ*), also known as a dielectric constant, is an ability of a medium to transmit an electric field [11]. Usually, the value of relative permittivity is considered as 81 [11,20], but in actual this parameter is complex valued and it further depends on the salinity of water, temperature and carrier frequency [21]. The dielectric permittivity in Arabian and Indian ocean costal lines has effectively been calculated and is estimated to be approximately *μ* = 81 [22].

#### 3.2.3. Electrical Conductivity of the Medium

The conductivity of medium (*δ*) is the overall effect over EM waves when they pass through the medium. It is calculated in Siemens per meter (S/m). When EM waves propagate through a conductive medium, they will reflect the EM waves. Conductivity of any medium depends on the number of ions present. When we consider water as a medium of propagation, which is conductive in nature, it will reflect EM waves. However, water medium itself can be categorized into three types with respect to its conductivity as shown in Table 3.

The conductivity of the Red Sea is 8 S/m, whereas it is only 2 S/m in the Arctic. Commonly, for sea water, the conductivity δ=4 S/m [4,22,23]. This is 400 times higher than the conductivity of freshwater, which is typically around 0.001 S/m [11]. If EM waves will propagate through such mediums, they will be attenuated. Thus, conductivity of a medium has a direct relationship to the propagation of EM waves, *i.e.*, with the increase in conductivity, attenuation will be increased and EM waves will travel less distance. The velocity of EM waves is also reduced because of the change of medium from air to water. The rate of reduction of EM waves is dependent on the relative dielectric permittivity $\epsilon_{r}$, the relative magnetic permeability $\mu_{r}$ and the electrical conductivity *δ* and given by:

(4) $$v=\frac{c}{\sqrt{\left(\epsilon_{r}\right)\left(\mu_{r}\right)\left(\frac{1+\sqrt{1+(\frac{\delta }{{\left(\omega \epsilon \right)}^{2}})}}{2}\right)}}$$

Due to the factors of water listed above, the fundamental physical behavior of EM waves changes in UW environments. We further extend our discussion on EM propagation in fresh and sea water.

### 3.3. EM Propagation in Freshwater

Freshwater is considered as a low-loss medium due to its low conductivity. The amount of turbidity is negligible. This enables EM waves to propagate in freshwater [24].

#### 3.3.1. Propagation Velocity of EM in Freshwater

The propagation speed of EM waves [11] can be expressed in Equation (5):

(5) $$c\approx \frac{1}{\sqrt{\mu \times \epsilon }}$$

where *ϵ* is the dielectric permittivity and *μ* is the magnetic permeability. The dielectric permittivity *ϵ* is further given as the product between permittivity in air ($\epsilon_{o}$) and relative permittivity ($\epsilon_{r}$) of a medium. The relative permittivity ($\epsilon_{r}$) is dimensionless quantity, which, in the case of water (fresh or saline) is about 81 [4].

#### 3.3.2. Absorption Losses in Freshwater

The amount of absorption loss is calculated using Equation (6) [7]:

(6) $$\alpha \approx \frac{\sigma }{2}\sqrt{\frac{\mu }{\epsilon }}$$

where *σ* is electrical conductivity, *ϵ* is electrical permittivity and *μ* is the permeability of the medium. It is clearly evident from Equation (5) and Equation (6) that the velocity of propagation and the absorption coefficient both are independent of the operating frequency.

### 3.4. EM Propagation in Seawater

Seawater is considered a high-loss medium due to the fact that it is highly conductive. The conductivity of seawater is almost 400 times greater compared to freshwater [4,25].

#### 3.4.1. Propagation Velocity of EM Waves in Seawater

Propagation in such a conductive medium makes it difficult for EM waves to propagate. Therefore, the propagation velocity is expressed in Equation (7) [7]:

(7) $$c\approx \sqrt{\frac{4\pi f}{\mu \epsilon }}$$

#### 3.4.2. Absorption Loss for EM in Seawater

The absorptive loss for EM waves in seawater is expressed in Equation (8) [7]:

(8) $$\alpha \approx \sqrt{f\mu \sigma \pi }$$

Equation (7) and Equation (8) clearly show that the velocity of propagation decreases while the absorption losses increase as EM waves propagate in seawater. Thus, aforementioned characteristics influence the EM wave propagation in underwater.

## 4. Experiments and Results

In this section, the experiments are performed in two different environments (Terrestrial and Underwater). Later, the path loss exponent is estimated for the two environments, and a detailed analysis is presented by comparing the overall path loss in the two environments. Moreover, the influence of different water conductivities over the velocity of EM waves is presented, which is based on analytical results. To conclude, results of the possible effects of different water conductivities creating absorption losses at 2.4 GHz and other radio frequencies are presented.

**Figure 2:**
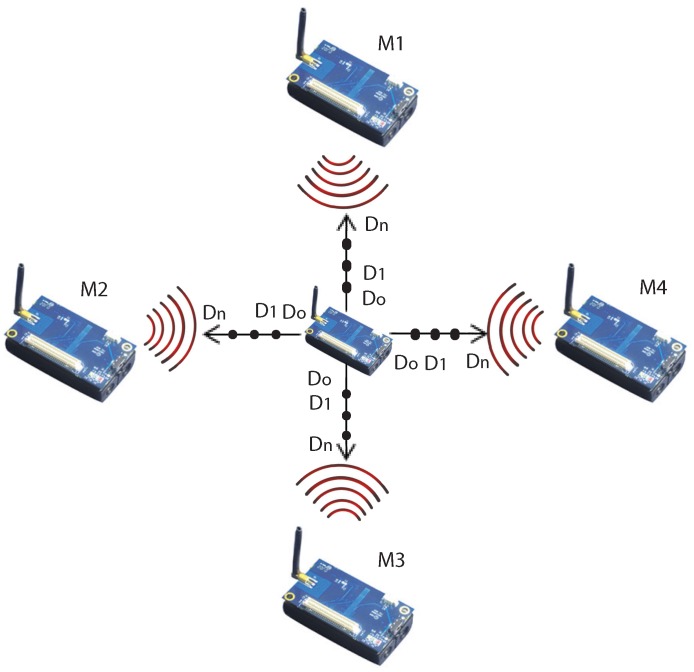
Methodology.

Therefore, the initial objective is to estimate the path loss exponent first in a terrestrial indoor environment, then in an underwater environment and provide a comparative analysis over the loss profile. Estimating losses in underwater is more challenging as compared to estimating losses in a terrestrial environment. Thus, a methodology needs to be designed which could be followed to carry out the experiments. It is important that methodologies adopted should give accurate and sensible results. To put devices underwater while transmitting radio signals is an intricate task. The methodology adopted is shown in Figure 2. The path loss is a function of Signal Strength received at the receiver from a discrete distance point. In order to acquire the RSS, we have used IRIS motes from Crossbow that works over a frequency band of 2.4 GHz. The IRIS mote configuration is shown in Table 4.

In order to estimate the RSS from the data packets, the methodology is devised in a manner that maximum samples must be taken to have a close approximation. Therefore, 4 IRIS motes (M1, M2, M3, M4) initially configured, transmit RSS data packets at 2.4 GHz from the particular distance to the Base station mote (Central mote) at the center as shown in Figure 2. The base station mote will receive those packets from the radio and will estimate RSS at that particular distance.

Figure 2 shows the methodology to acquire RSS data that can be adopted for both environments, in the Terrestrial Indoor Environment as well as the Underwater Environment. In the experiment, four IRIS motes M1, M2, M3 and M4 are positioned at $0^{\circ }$, $90^{\circ }$, $180^{\circ }$ and $270^{\circ }$ with respect to the base station mote. All motes initially are located at one reference distance ($d_{o}$) with respect to base station. All four IRIS motes will transmit data packets from their locations and distinct distance points, which will be received by the base station mote. The base station mote will extract the RSS from those packets and will indicate the estimated value of RSS.

This experiment will be periodically repeated at different distance points with respect to the base station in all four directions, in order to sample the power values at discrete points to estimate the path loss exponents for both environments.

### 4.1. Scenario 1: Estimating Path Loss in Terrestrial Indoor Environments

In order to estimate the Path Loss in an Indoor Environment, the very first experiment was setup in Seminar Hall of the Department of Telecommunication Engineering at Mehran University of Engineering & Technology (MUET), Jamshoro, Pakistan. Following the experimental setup as shown in Figure 2, four IRIS motes (M1, M2, M3 and M4) are used. M1 placed at $0^{\circ }$ , M2 at $90^{\circ }$, M3 at $180^{\circ }$ and M4 placed at $270^{\circ }$ with respect to the base station. The antenna orientations of the IRIS transmitters were at $0^{\circ }$ vertical in line with each other and also with the base station’s antenna.

The reference distance is taken as 0.3 m. As per the experimental setup proposed in Section 4, distance is increased with a difference of 0.3 m up to 1.5 m distinctive points.

**Figure 3:**
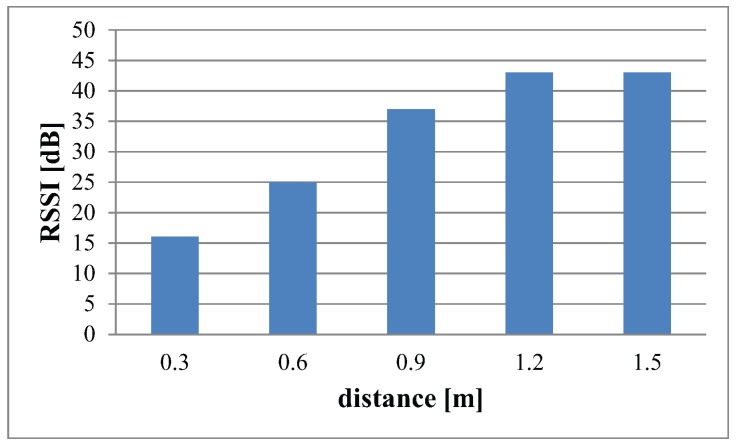
RSS values at different distances in indoor environments.

These results are obtained from RSS at different distances in an indoor environment. Figure 3 illustrates the overall path loss plotted from the RSS data collected in terrestrial indoor environments. With the help of a curve fitting tool in MATLAB, the best possible approximation of linear regression is calculated. Mathematically, this linear approximation is given as:

(9) y=18×x+37

The blue graph depicts the averaged RSSI from 0.3 m to 1.5 m. From the above Equation (9), we get the value for slope of the line m=18. Therefore, from the relation stated in Section 3.1, we calculate the value for path loss exponent n=1.8. This verifies the path loss exponent for a terrestrial indoor environment as mentioned in Table 2. Figure 3 is the bar graph illustrating the RSS measured at each distance.

### 4.2. Scenario 2: Estimating Path Loss in Underwater Environments

To estimate the path loss in underwater environments, the second experiment was conducted in a 25 m wide and 50 m long swimming pool whose depth was from 3 to 16 ft. IRIS motes were put 1 ft under the water to estimate the path loss exponents and as well as overall path loss attenuation in underwater environments operating at 2.4 GHz. The conductivity of water in the experimented medium was 0.001 S/m. Therefore, the water is categorized as freshwater. To calculate the path loss underwater, a similar approach is followed as proposed in Section 4. The placement of nodes is similar to what has been illustrated in Section 4.1. The base station is connected with the PC to project the real-time results. The antenna orientation of the IRIS motes is kept $0^{\circ }$ vertical in line with each other and with the base station antenna. To make sure that the motes are not damaged, IRIS motes are kept in rectangular cubicles that are water proof. The boundaries of the rectangular cube are kept at a distance greater than λ/2 of the highest frequency (upper frequency) of the selected channel of 2.4 GHz. This is important because, in LOS communication, if any object or obstacle comes under λ/2, distance would make the small-scale fading factors significant enough to create large effects over the large-scale fading.

**Figure 4:**
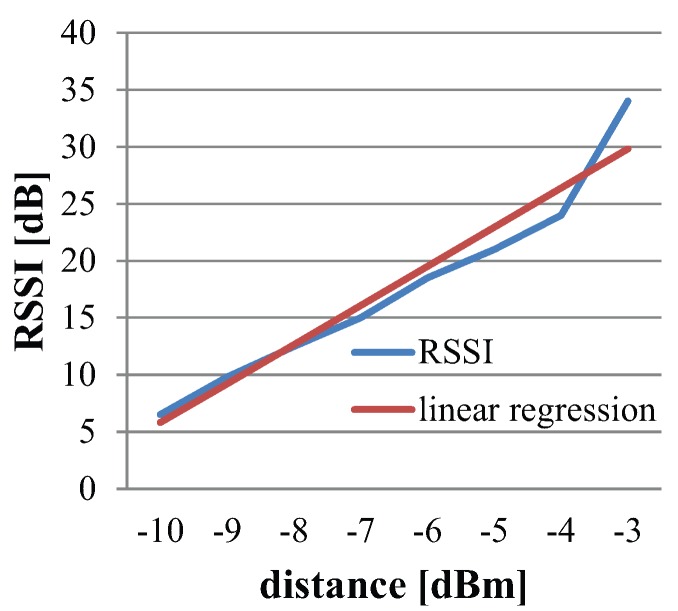
Estimating the path loss exponent underwater environment.

Thus, the results obtained from the experiment are shown in Figure 4. Interestingly, when IRIS motes were put inside water, a drastic change was observed in signal transmission and reception due to the sudden change in medium from air to water. Due to severe attenuation in underwater medium, the reference distance is kept 0.1 m. The distance is increased with the step size of 0.1 m up to 0.5 m.

The results shown in Figure 4 illustrate the overall path loss obtained from the RSS data collected in underwater environments, specifically in freshwater. The best possible approximation of linear regression is shown with the red graph. Mathematically, this linear approximation is given as:

(10) y=35×x+12

**Figure 5:**
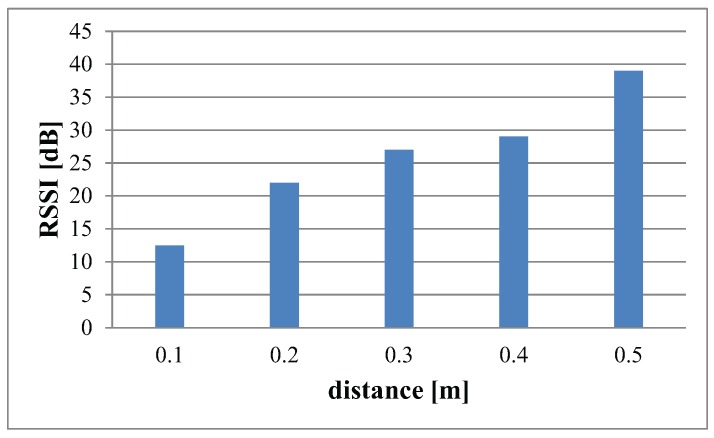
RSS at different distances in underwater environment.

The blue graph depicts the averaged RSS from 0.1 m to 0.5 m. From Equation (10), the value of the slope of the line, *i.e.*, m=35 is calculated. Thus, the path loss exponent will be n=3.5. This verifies that the path loss exponent for an underwater environment is as mentioned in Table 2. Figure 5 is the bar graph of data illustrating the RSS measured at each distance.

### 4.3. Comparative Analysis of Underwater Environments and Terrestrial Indoor Environments

By comparing the results, we can analyze the differences in signal degradation when a signal propagates in terrestrial indoor environments and when a signal propagates in underwater environments. From the results, the path loss exponent for indoor environments is 1.8, and the path loss exponent calculated for underwater environments is 3.4. This shows that when the signal propagates in an indoor environment, it degrades with the order of 1.8 and when the signal propagates underwater, the signal degrades with the order of 3.4. Underwater signal degradation is far greater than terrestrial degradation due to the channel characteristics.

**Figure 6:**
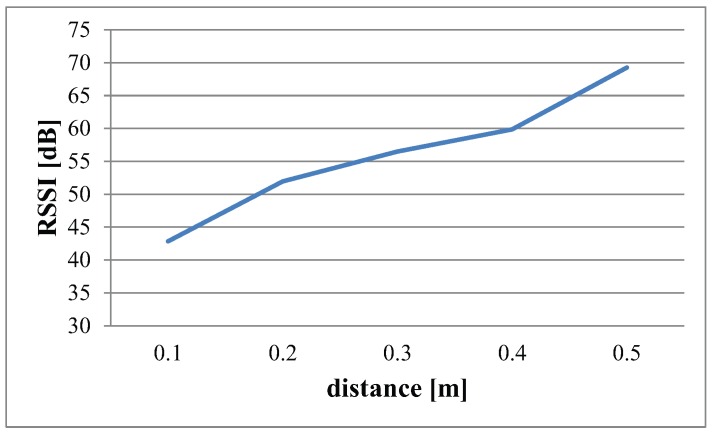
Overall path loss in underwater environment.

Figure 6 shows the overall path loss in underwater environments. The path loss calculated is 26.5 dB from 0.1 m to 0.5 m. Thus, when EM signal propagate underwater, specifically in freshwater, they undergo a signal loss of around 26.5 dBs with a path loss exponent of 3.4.

### 4.4. Velocity of EM Wave Underwater

**Figure 7:**
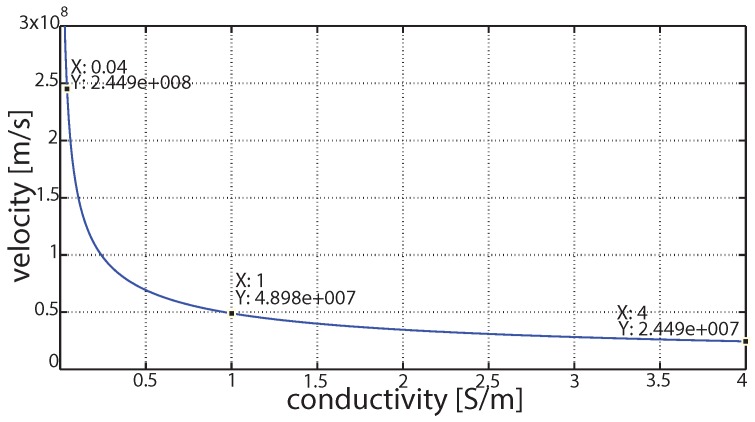
Velocity of EM waves at different conductivities of water.

The velocity of sound underwater is 1500 m/s, which is slow when compared to the velocity of EM signals in the same medium. Thus, EM waves are faster than acoustic waves, hence yielding less propagation delays and enabling fast communication. Figure 7 shows the velocity of propagation when an EM signal propagates inside water.

Figure 7 evidently shows a significant decrease in the velocity of EM waves. This holds true because the quality of water is inconsistent everywhere. At every stage of the water flow, the water quality differs, and this difference is due to the amount of solid particle concentration in water that contributes significant changes in the water conductivity. As the water quality decreases, there is a significant increase in conductivity of water that is commonly found. A high conductive medium reflects the EM waves more than the low conductive medium, making it difficult for the EM wave to travel through it. Table 3 elaborates the possible water conductivities, and, based on that, categorizes the nature of water. Thus, Figure 7 shows how the velocity of EM waves gets affected as water conductivity increases, which has a significant impact over EM waves.

### 4.5. Absorption Losses in 2.4 GHz in an Underwater Environment

Whenever EM signals propagate underwater, the EM radiation gets absorbed and the signal strength decreases strongly leading to absorption loss. We estimated the amount of absorption losses when 2.4 GHz is transmitted underwater specifically in freshwater.

**Figure 8:**
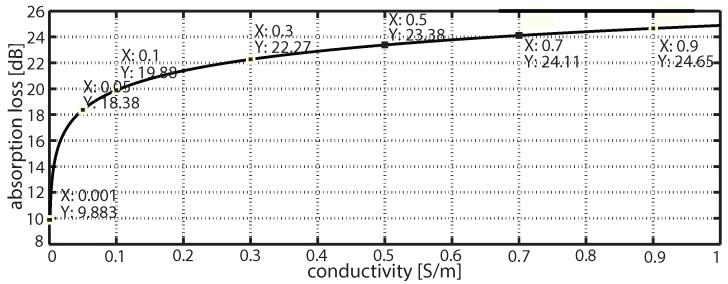
Absorption loss in 2.4 GHz in freshwater conductivity.

**Figure 9:**
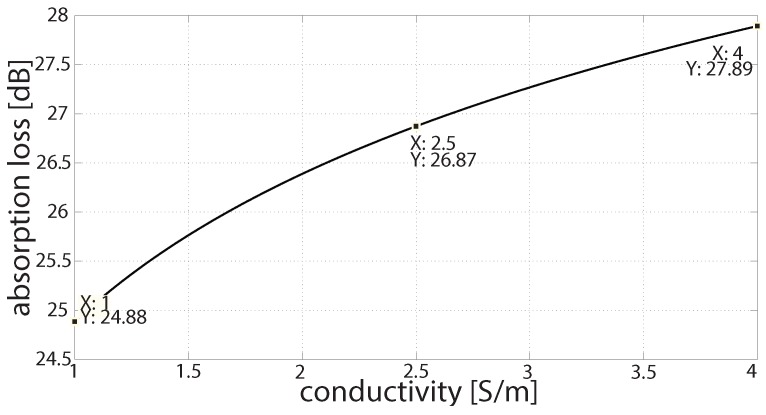
Absorption loss in 2.4 GHz in river water and seawater conductivities.

Figure 8 shows the absorption loss as 2.4 GHz signal propagates in freshwater. The conductivities of freshwater range from 0 S/m to less than 1 S/m. As the signal propagates and the water conductivity increases, the absorption losses are also increased. These losses become more significant when the water conductivity increases. Thus, Figure 9 shows the absorption losses at 2.4 GHz in river water and seawater conductivities.

From Figure 8 and Figure 9, the following observations have been made. The absorption losses are about 9.8 dB to 24 dB in freshwater, when water conductivity ranges from 0 S/m to 0.9 S/m at 2.4 GHz. The absorption losses are increased to 19 dB to 24 dB in river water conductivities at 2.4 GHz, respectively. These absorption losses increase to up to 25 dB to 30 dB in seawater conductivities at 2.4 GHz. The losses at 2.4 GHz are extremely high. Accordingly, we test MHz radios and estimate the losses in different water conductivities.

### 4.6. Absorption Losses in MHz Radio Bands at Different Conductivities of Water

**Figure 10:**
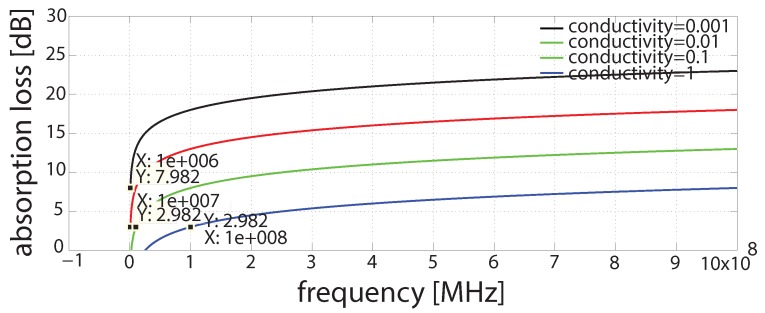
Absorption loss for MHz frequency (radios) at different conductivities of water.

The results shown in Figure 10 show the range of radio frequencies whose losses are under 3 dB.

The results in Figure 10 show the absorption losses for MHz radios in various water conductivities. All of the results are summarized in the Table 5.

Thus, for absorption losses to be less than 3 dBs at water conductivity of 0.001 S, the radios ranging from $1\times 10^{8}$ Hz or greater can be used. Similarly, for absorption losses to be less than 3 dB at water conductivity of 0.01 S, the radios ranging from $1\times 10^{7}$ Hz or greater can be used. In addition, for absorption losses less to be than 3 dB at water conductivity of 0.001 S, the radio ranging from $1\times 10^{6}$ Hz or greater can be used.

## 5. Conclusions

The paper highlights the characteristics of the channel and possible effects over the EM frequencies, specifically over the 2.4 GHz ISM frequency band. After the deliberation of EM properties of the medium, we tested 2.4 GHz in freshwater and estimated the path loss. It is concluded that path loss inside fresh water at 2.4 GHz is 1.6 times greater than the path loss in the terrestrial indoor environment using the same frequency. Furthermore, from the results of velocity of propagation of 2.4 GHz in underwater, it is concluded that velocity of EM decreases as water quality degrades. Propagation of the speed of sound underwater is 1500 m/s; however, sound waves cause a significant delay in the overall transmission. EM waves are faster than acoustics even underwater, thus, EM waves can be considered real-time applications. In addition, from the results of absorption losses for 2.4 GHz when simulated underwater at different medium conductivities, it is concluded that the absorption losses increase as water conductivity increases. Specifically, absorption losses are about 9 dB to 19 dB in freshwater conductivity at 2.4 GHz, the absorption losses are about 19 dB to 24 dB in river water conductivity at 2.4 GHz, and absorption losses are about 25 dB to 30 dB in seawater at 2.4 GHz.

Moreover, we verified a subset of frequencies for which the absorption losses are less than 3 dB for different water conductivities. Therefore, for absorption losses less than 3 dB at water conductivity of 0.001 S, the radios ranging from $1\times 10^{8}$ Hz or greater can be used. Similarly, for absorption losses less than 3 dB at water conductivity of 0.01 S, the radios ranging from $1\times 10^{7}$ Hz or greater can be used. In addition, for absorption losses less than 3 dB at water conductivity of 0.001 S, the radios ranging from $1\times 10^{6}$ Hz or greater can be used. Thus, this paper proposes EM waves for UWSNs. The results presented in this paper highlight the significance of EM waves for UW communications. EM waves have significance when considering shallow water communication and real-time applications. There are many RF frequencies that give less attenuation in certain conditions. In fresh and river water conditions, EM out performs acoustics in terms of bandwidth.

## Figures and Tables

**Table 1:** Underwater wireless communication systems.

Carrier	Transmission Distance	Bandwidth	Mode of Communication
Accoustics	1000 km	<1 kHz	Non Line of Sight
Radio	<1 m	1 MHz	Non Line of Sight
Optical	<10 m	1 GHz	Line of Sight

**Table 2:** Path loss exponent for different environments.

Environment	Path Loss Exponent “n”
Free Space	2
Urban Area	2.7 to 3.5
Sub Urban Area	3 to 5
Indoor (Line of Sight)	1.6 to 1.8
Underwater (Line of Sight)	2 to 4

**Table 3:** Water conductivity.

Water	Conductivity Values
Freshwater	0 ≤ *δ* <1
Riverwater	1 ≤ *δ* <2
Seawater	*δ*≥ 2

**Table 4:** IRIS Mote Configuration.

Device	Parameters	Value
IRIS MOTES	Default Power (Raw Value)	0
	Default Power (dBm)	3
	Default Power (Watts)	11.9 mW
	Default Channel	11
	Upper Cut-off Frequency	2.405 GHz
	Lower Cut-off Frequency	2.410 GHz

**Table 5:** Absorption Losses for MHz Radios at Different Conductivities of Water.

Conductivity Values (*δ*)	Frequency Ranges (Hz)	Estimated Losses (dB)
0.001 S	$10^{8}$	2.98
0.01 S	$10^{7}$	2.98
0.1 S	$10^{8}$	2.98

## References

[1] Guo Z., Li Z., Hong F. USS-TDMA: Self-stabilizing TDMA Algorithm for Underwater Wireless Sensor Network. Proceedings of the International Conference on Computer Engineering and Technology.

[2] Dario I., Akyildiz I., Pompili D., Melodia T. (2005). Underwater Acoustic Sensor Networks: Research Challenges. Ad Hoc Netw..

[3] Felemban E., Shaikh F.K., Qureshi U.M., Sheikh A.A., Qaisar S.B. (2015). Underwater Sensor Network Applications: A Comprehensive Survey. Int. J. Distrib. Sens. Netw..

[4] Chaitanya D., Sridevi C., Rao G. Path loss analysis of underwater communication systems. Proceedings of the IEEE Students’ Technology Symposium (TechSym).

[5] Ramanathan N., Harmon T., Balzano L., Estrin D., Hansen M., Jay J., Kaiser W., Sukhatme G. Designing Wireless Sensor Networks as a Shared Resource for Sustainable Development. Proceedings of the International Conference on Information and Communication Technologies and Development.

[6] Preisig J. (2007). Acoustic Propagation Considerations for Underwater Acoustic Communications Network Development. SIGMOBILE Mob. Comput. Commun. Rev..

[7] Lloret J., Sendra S., Ardid M., Rodrigues J. (2012). Underwater Wireless Sensor Communications in the 2.4 GHz ISM Frequency Band. Sensors.

[8] Cui J., Kong J., Gerla M., Zhou S. (2006). The challenges of building mobile underwater wireless networks for aquatic applications. IEEE Netw..

[9] Giles J.W., Bankman I.N. Underwater optical communications systems. Part 2: Basic design considerations. Proceedings of the MILCOM 2005—IEEE Military Communications Conference.

[10] Sehgal A., Tumar I., Schonwalder J. Variability of available capacity due to the effects of depth and temperature in the underwater acoustic communication channel. Proceedings of the OCEANS 2009—EUROPE.

[11] Hattab G., El-Tarhuni M., Al-Ali M., Joudeh T., Qaddoumi N. (2013). An Underwater Wireless Sensor Network with Realistic Radio Frequency Path Loss Model. Int. J. Distrib. Sens. Netw..

[12] Che X., Wells I., Dickers G., Kear P., Gong X. (2010). Re-evaluation of RF Electromagnetic Communication in Underwater Sensor Networks. IEEE Commun. Mag..

[13] Nowsheen N., Benson C., Frater M. A high data-rate, software-defined underwater acoustic modem. Proceedings of the OCEANS 2010 MTS/IEEE SEATTLE.

[14] Wells I., Davies A., Che X., Kear P., Dickers G., Gong X., Rhodes M. Node pattern simulation of an undersea sensor network using RF electromagnetic communications. Proceedings of the International Conference on Ultra Modern Telecommunications Workshops.

[15] Sendra S., Lamparero J.V., Lloret J., Ardid M. (2012). Study of the optimum frequency at 2.4 GHz ISM band for underwater wireless ad hoc communications. Ad-hoc, Mobile, and Wireless Networks.

[16] Rappaport T. (2001). Wireless Communications: Principles and Practice.

[17] Stojanovic M. (2006). On the Relationship Between Capacity and Distance in an Underwater Acoustic Communication Channel. Proceedings of the 1st ACM International Workshop on Underwater Networks.

[18] Palmeiro A., Martin M., Crowther I., Rhodes M. Underwater radio frequency communications. Proceedings of the OCEANS 2011 IEEE—Spain.

[19] Sadiku M. (2014). Elements of Electromagnetics.

[20] Al-Shamma’a A., Shaw A., Saman S. (2004). Propagation of electromagnetic waves at MHz frequencies through seawater. IEEE Trans. Antennas Propag..

[21] Somaraju R., Trumpf J. (2006). Frequency, Temperature and Salinity Variation of the Permittivity of Seawater. IEEE Trans. Antennas Propag..

[22] Joshi A.S., Deshpande S.S., Kurtadikar M.L. (2012). Dielectric properties of north Indian ocean seawater at 5 GHz. Int. J. Adv. Eng. Technol..

[23] Hunt K., Niemeier J., Kruger A. RF communications in underwater wireless sensor networks. Proceedings of the IEEE International Conference on Electro/Information Technology (EIT).

[24] Sendra S., Lloret J., Rodrigues J.J., Aguiar J.M. (2013). Underwater wireless communications in freshwater at 2.4 GHz. IEEE Commun. Lett..

[25] Anguita D., Brizzolara D., Parodi G. Optical communication for Underwater Wireless Sensor Networks: A VHDL-implementation of a Physical Layer 802.15.4 compatible. Proceedings of the OCEANS 2009—EUROPE.

